# Artificial Intelligence in Pulmonary Hypertension: Current State and Future Prospects

**DOI:** 10.31083/RCM48114

**Published:** 2026-08-21

**Authors:** Zechen Li, Xiaowei Xu, Jiahong Li, Yushen Fang, Yinru He, Jiaqing Tang, Zhuyang Yang, Huiru Xie, Haiyun Yuan

**Affiliations:** ^1^Department of Cardiovascular Surgery, Guangdong Cardiovascular Institute, Guangdong Provincial People’s Hospital (Guangdong Academy of Medical Sciences), Southern Medical University, 510080 Guangzhou, Guangdong, China; ^2^Guangdong Provincial Key Laboratory of South China Structural Heart Disease, 510080 Guangzhou, Guangdong, China; ^3^Guangdong Provincial People’s Hospital (Guangdong Academy of Medical Sciences), Southern Medical University, 510080 Guangzhou, Guangdong, China

**Keywords:** pulmonary hypertension, artificial intelligence, machine learning, deep learning, cardiac imaging techniques, diagnostic imaging

## Abstract

Pulmonary hypertension (PH) remains diagnostically challenging due to the associated non-specific symptomatology and frequent diagnostic delays, both of which contribute to increased morbidity and mortality. Meanwhile, right heart catheterization is the diagnostic gold standard; nonetheless, the invasive nature and limited accessibility of this technique limit its routine use, particularly in resource-constrained settings. This review evaluates computational approaches that may enhance PH diagnosis through advanced analysis of cardiovascular imaging data. We conducted a comprehensive literature review focusing on computer-assisted diagnostic methods in PH across multiple imaging modalities, including electrocardiogram, chest X-ray, echocardiography, cardiac magnetic resonance (CMR) imaging, and cardiac computed tomography (CCT). Eligible studies were analyzed for diagnostic performance, clinical applicability, and methodological rigor. Preliminary studies have demonstrated promising performance in detecting early or subclinical PH phenotypes across various imaging platforms. Advanced imaging modalities benefit from automated segmentation and quantitative analysis, and CMR- and CT-based approaches demonstrate high diagnostic accuracy. Current artificial intelligence (AI) models face significant challenges related to clinical interpretability, external validation across diverse populations, and seamless integration into existing diagnostic workflows. Most studies are based on single-center retrospective cohorts, underscoring the need for multicenter prospective validation. To address these challenges, future research must prioritize advancing methodological transparency through explainable AI (XAI) and ensuring data privacy via federated learning. Crucially, the next phase of innovation lies in synergistic multimodal fusion (MMF) architectures that synthesize heterogeneous data—ranging from imaging to hemodynamics—to enhance phenotypic precision. Furthermore, leveraging large language models (LLMs) for computational phenotyping from electronic health records offers a scalable solution for identifying undiagnosed patients. Finally, realizing clinical translation requires rigorous multicenter prospective validation and seamless integration into existing workflows to ensure these tools effectively support decision-making in real-world practice. While computational approaches in PH diagnostics show promise for improving early detection and diagnostic accuracy, significant challenges remain before widespread clinical adoption. Future development should prioritize multicenter validation, standardized frameworks integrating multi-parametric imaging with clinical biomarkers, and transparent methodologies to support clinical decision-making. The integration of these computational tools into conventional diagnostic pathways may enhance PH management through earlier detection and more precise risk stratification.

## 1. Introduction

Pulmonary hypertension (PH) is a pathological condition that can be linked to a variety of clinical situations and is associated with a range of cardiovascular diseases. PH is hemodynamically defined by a mean pulmonary artery pressure (mPAP) greater than 20 mmHg, resulting in increased right ventricular (RV) strain and frequently leading to RV failure. PH, affecting ~1% of the global population, is often diagnosed at advanced stages due to nonspecific symptoms, leading to delayed treatment and poor prognosis [1,2]. Since the early symptoms of PH are not distinct, it is difficult for clinicians to diagnose accurately, which may lead to the delay of patients’ illness. Due to the nonspecific nature of early symptoms, such as dyspnea, and the relative rarity of PH compared to other conditions with similar manifestations, diagnostic delays are common, often lasting more than two years. These delays are associated with an increased risk of morbidity and mortality [3,4]. Therefore, early disease recognition and effective screening tools are indispensable. Early detection and referral to a specialist PH center are crucial for establishing targeted management, which can relieve symptoms and optimize prognosis. Furthermore, PH is a heterogeneous condition, and accurate patient phenotyping is crucial for identifying the underlying cause and developing the appropriate therapeutic strategy [5].

Right heart catheterization (RHC) is considered the gold standard for both diagnosing and classifying PH [1], offering direct measurement of pulmonary artery pressure and vascular resistance. However, its invasiveness and reliance on specialized infrastructure limit its routine application, particularly in primary care and resource-limited settings. Transthoracic echocardiography (TTE) is commonly recommended as the first-line tool for screening, but its use is inconsistent in early-stage PH, and key parameters—such as right ventricular (RV) size, function, and indirect echocardiographic signs—are frequently underreported or omitted. The diagnostic evaluation of PH often requires a multimodal imaging approach, incorporating electrocardiogram (ECG), chest X-ray (CXR), echocardiography, cardiac magnetic resonance (CMR) imaging, and cardiac computed tomography (CCT), each contributing complementary information. Interpretation of these diverse and complex datasets, however, requires substantial clinical expertise and is susceptible to interobserver variability.

Given the wide array of comprehensive data sets, ranging from complex disease diagnosis and management to multimodal imaging, PH presents an ideal area for the application of artificial intelligence (AI). By integrating and analyzing multimodal data, AI can identify patterns and insights that may be overlooked by human clinicians, enhancing diagnostic precision and clinical decision-making [6]. In particular, advancements in Machine Learning (ML) and Deep Learning (DL) have facilitated the development of more efficient, non-invasive diagnostic tests, offering new possibilities for early detection and monitoring.

Advancements in computer science and continual improvements in hardware infrastructure have led to an exponential growth in clinical data. As multimodal data accumulates at an unprecedented rate, it becomes increasingly challenging to analyze and interpret using conventional methods. ML has emerged as a powerful solution to this problem. At its core, ML represents the interaction between large-scale datasets and specific algorithmic frameworks designed to identify patterns and make predictions within complex data environments. Compared to traditional statistical approaches, ML methods are more exploratory and hypothesis-generating in nature. They also demonstrate superior performance in handling high-dimensional datasets, making them particularly well-suited for modern medical data analysis [7]. Zack et al. [8] demonstrated that ML, specifically random forest models, outperformed traditional logistic regression in predicting outcomes such as rehospitalization and cardiovascular mortality, highlighting ML’s superior predictive power. In PH, ML algorithms effectively manage diverse data types, including clinical, imaging, and multi-omics data. Emily Kogan et al. [9] developed an ML tool that accurately predicts PH diagnoses, even in patients without characteristic symptoms, using a large control population to mitigate overfitting. This model showed high accuracy (area under the curve [AUC] 0.92) in predicting PH diagnoses up to 18 months in advance and also demonstrated strong performance in identifying specific PH subtypes.

Various ML methods are capable of identifying both linear and intricate nonlinear relationships between variables, as they make fewer assumptions about the underlying data compared to traditional statistical models [10]. DL, a subset of ML, is inspired by the structure of the human brain and utilizes artificial neural networks to model intricate relationships between input data and corresponding outcomes or labels. These models are particularly effective in imaging tasks, where the complexity of input features, such as pixels and voxels, plays a significant role [11,12]. DL, which draws inspiration from neural network architecture, is a powerful method that can be applied to both supervised and unsupervised learning tasks. DL utilizes a more fluid data flow approach. This allows it to model complex relationships with greater flexibility. In particular, DL has been proven to outperform traditional methods when handling high-dimensional, unstructured data, such as medical images [13].

Applying AI technologies to comprehensive PH datasets could help optimize diagnostic strategies tailored to individual patient needs. Thus, to address the specific needs of PH patients, it is crucial to implement strategic initiatives that advance AI-driven research and its clinical applications. This review aims to synthesize current AI applications in PH diagnosis, address technical challenges, and propose future directions for integrating AI into clinical workflows.

## 2. Materials and Methods

A literature search was conducted in PubMed for publications from 2000 to 2025, using MeSH terms and free-text keywords such as “Artificial Intelligence”, “Machine Learning”, “Deep Learning”, “Pulmonary Hypertension”, “Pulmonary Arterial Hypertension”, “Diagnosis”, “Classification”, and “Cardiology”, combined with Boolean operators to refine the search. Studies were included if they focused on AI-based methods for PH diagnosis, classification, or prognosis; used cardiovascular imaging modalities (ECG, CXR, echocardiography, CMR, or computed tomography [CT]); incorporated clinical, hemodynamic, or multi-omics data; and were peer-reviewed original research, systematic reviews, or clinical trials published in English with quantitative performance metrics. Conference abstracts and preprints were eligible if they provided sufficient methodological and outcome data for assessment. Studies were not required to include an independent external validation cohort. Validation approaches were classified as internal validation, including cross-validation or bootstrap resampling, or external validation using an independent dataset. The literature was analyzed for AI applications in improving diagnostic precision, phenotypic classification, and clinical decision support, with data extraction focusing on study design, patient populations, AI methods, imaging protocols, performance metrics, and clinical validation. Quality was assessed using established criteria for diagnostic accuracy studies and AI research methodologies.

To assess the risk of bias in the included studies, we used the PROBAST (Prediction model Risk of Bias Assessment Tool). A summary of the PROBAST scoring for specific studies, detailing their risk of bias across key domains, is provided in Appendix Table 6.

## 3. Intelligent Diagnosis of Pulmonary Hypertension

The basic concepts related to AI have been introduced above. This section will introduce the traditional diagnostic methods of PH in sections. On this basis, the application of existing AI for intelligent diagnosis of PH will be introduced in detail.

### 3.1 Introduction of PH Diagnosis

The primary goal of classifying PH clinically is to group related conditions based on shared pathophysiological mechanisms, clinical features, hemodynamic profiles, and treatment strategies. PH is characterized by a mPAP exceeding 20 mmHg at rest. Regarding to the Hemodynamic, we can briefly defined PH into three groups, Precapillary PH, IpcPH (isolated postcapillary pulmonary hypertension) and CpcPH (combined post- and precapillary pulmonary hypertension). PH is clinically classified into five distinct groups: Group 1 – Pulmonary arterial hypertension (PAH), Group 2 – PH related to left heart disease (LHD), Group 3 – PH linked to lung diseases and/or hypoxia, Group 4 – PH resulting from pulmonary artery obstructions, and Group 5 – PH with uncertain or multifactorial causes. PH is most frequently associated with diseases affecting the left side of the heart (WHO Group 2 PH) and respiratory conditions characterized by hypoxia (WHO Group 3 PH), together accounting for approximately 90% of global PH cases. In contrast, PAH (WHO Group 1) and PH caused by pulmonary artery obstruction (WHO Group 4) are less common, each representing less than 5% of all PH diagnoses. Despite their rarity, these two groups are central to clinical PH evaluations due to the availability of specific treatment strategies tailored for patients in both categories [1].

RHC remains the gold standard for diagnosing and classifying PH. However, due to its invasive nature, high cost, and limited availability in primary or lower-tier healthcare settings—as well as associated risks and contraindications—RHC is not suitable for routine screening or early evaluation in large patient populations. Consequently, non-invasive modalities such as electrocardiogram, chest radiography, echocardiography, computed tomography (CT), and CMR imaging are more commonly employed for initial assessment and monitoring. In the following sections, we summarize recent advances in these non-invasive diagnostic techniques and explore how AI can enhance their utility in PH screening, diagnosis.

### 3.2 Electrocardiogram-Based AI Applications

ECG is a common clinical test that can be a valuable tool in diagnosing PH. Abnormal ECG findings can indicate right heart issues, raise suspicion of PH, and provide prognostic information for high-risk patients. ECG has been explored for population-based screening for PH, as it is widely available, inexpensive, non-invasive, and provides consistent results with little variability across different medical systems [14].

#### 3.2.1 Feature-Based and Waveform-Driven Approaches for Screening, Diagnosis, and Classification

ECG abnormalities may raise suspicion of PH, deliver prognostic information, and detect arrhythmias and signs of LHD. Traditional ECG interpretation relies on manual identification of features such as P waves and QRS complexes. In contrast, AI models can automatically extract latent features related to PH from raw ECG signals, such as signs of right heart strain, right ventricular hypertrophy, and electrical axis deviation. In adults with clinical suspicion of PH (e.g. unexplained dyspnoea on exertion), right axis deviation has a high predictive value for PH [15]. In 2019, Sawada et al. [16] demonstrated that nationwide ECG-based screening in schoolchildren could identify pediatric patients with idiopathic/heritable PAH who exhibited severe hemodynamic impairment despite mild symptoms and preserved right heart function. Although clinician-interpreted ECG enabled detection of otherwise unrecognized cases, traditional ECG criteria showed limited diagnostic accuracy, underscoring the risk of underdiagnosis. These limitations highlight the potential value of AI-enabled deep ECG analysis, which may enhance detection of elevated pulmonary arterial pressure through comprehensive waveform evaluation.

However, although similar findings have been reported, current ECG patterns based on traditional markers are not diagnostic for PH. The sensitivity and specificity of these rudimentary algorithms are limited, which may result in considerable underdiagnosis or misdiagnosis of PH. In contrast, deep ECG analysis, which involves detailed waveform evaluation, is well-suited for integration with AI. AI can be trained to detect subtle waveform variations that are often unnoticed by human clinicians, allowing for high-throughput analysis across large patient populations. This approach has the potential to uncover subtle ECG patterns that correlate with variations in clinical outcomes, a technique that has proven valuable in many other diseases [17,18].

DuBrock et al. [19] retrospective analysis was conducted on ECG data from a large cohort of patients suspected of having PH, based on RHC. A convolutional neural network, the PH Early Detection Algorithm (PH-EDA), was used to detect subtle ECG signs commonly seen in PH, focusing on right heart abnormalities such as right ventricular hypertrophy and right axis deviation. The model’s performance, tested on pre-diagnosis ECGs, showed an AUC of 0.87 within one month of diagnosis and 0.73 up to five years before clinical confirmation of PH. This method effectively identified PH cases based on either RHC or echocardiographic evidence, though it did not differentiate between disease phenotypes. Such a large-scale, automated screening approach holds promise for identifying early-stage PH, particularly PAH, even before symptoms appear. Early detection could address key issues in PH management, including risk factor modification, optimal treatment timing, identifying patients at high risk of rapid progression, and defining low-risk subgroups.

Kwon et al. [20] used a DL AI algorithm focused on the QRS complex, particularly the S-wave, to identify a strong correlation with PH in patients with a narrow QRS. Their results showed that PH patients had longer QRS durations and greater right axis deviation compared to those without PH. ECG data were from the University of California, San Francisco (2012–2019). A convolutional neural network trained on 12-lead ECG voltage data from a large UCSF cohort demonstrated strong performance for PH detection, with robust discrimination across multiple PH subtypes. Notably, diagnostic accuracy remained stable when the model was applied to ECGs acquired up to two years prior to clinical diagnosis, supporting its potential role in early disease identification. Aras et al. [21] developed a DL model based on raw 12-lead ECG waveforms to identify PH using either RHC or echocardiographic criteria as reference standards. The model demonstrated robust discrimination across multiple PH subtypes and retained meaningful diagnostic performance when applied to ECGs obtained up to two years prior to clinical diagnosis, highlighting the potential of ECG-based AI for early risk stratification and reduction of diagnostic delay in PH. These findings indicate that this DL-based ECG algorithm can accurately detect PH and its subtypes, potentially reducing diagnostic delays.

#### 3.2.2 Multimodal Data Integration and Wearable Technology

Liu et al. [22] developed a DL model (DLM) combining paired ECG and CXR data to detect elevated pulmonary artery pressure (ePAP). The model demonstrated high diagnostic performance across internal and external datasets, with combined AUCs of 0.8644 and 0.8734, respectively, and negative predictive values exceeding 98%. Beyond detection, it effectively predicted future risks of left ventricular dysfunction and cardiovascular mortality. This highlights the potential of multimodal AI models to enhance early PH identification, reduce diagnostic bias, and support timely

Nemati et al. [23] conducted a multicenter validation framework (CADLAD/IDENTIFY/IDENTIFY-PH/RADPH, N = 456 including 252 PH-positive and 204 PH-negative cases) established an ML algorithm leveraging multimodal photoplethysmography (PPG) and oscillometric waveform analysis (8 kHz sampling via CorVista). Through a three-stage computational pipeline: (1) Feature Engineering: 3,298 handcrafted features spanning temporal, spectral, time-frequency, and phase-space domains, reduced to 217 significant predictors via univariate screening (*t*-test/AUC/mutual information); (2) Model Architectures: Elastic Net-Random Forest hybrid classifier integrating linear/nonlinear relationships; (3) Robust Validation: 100-iteration bagged ensembles with five-fold stratified cross-validation. The algorithm demonstrated consistent diagnostic performance (mPAP ≥25 mmHg: AUC 0.93, sensitivity 87%, specificity 83%; ESC 2022 thresholds [mPAP ≥21 mmHg]: AUC 0.91, sensitivity 80%) across demographic subgroups (male AUC 0.95 vs. female 0.90; elderly ≥65 AUC 0.89 vs. younger 0.94) and PH subtypes. This scalable, non-invasive solution addresses critical gaps in settings lacking echocardiography or RHC capabilities, with future directions exploring combinatorial diagnostics with other cardiovascular biomarkers.

Furthermore, recent studies have shown that PH can be detected and predicted using single-lead wearable devices, especially those with right precordial leads (V2-V4), coupled with AI algorithms. This method, alongside traditional 12-lead ECG, holds promise for efficient PH screening in at-risk patients. Wearable technologies such as watches and patches could serve as an effective tool for PH prediction, offering a cost-effective and accessible solution, particularly in regions with limited healthcare resources [24]. Table 1 (Ref. [16,19,20,23]) summarizes the key findings related to the application of AI in ECG as discussed in this section.

**Table 1.** **Summary of AI in ECG applications in PH**.

Author (Year)	Population & PH-group	Threshold & Reference standard	Study design & Validation	Model	Key findings& Performance	Limitations
Sawada et al. (2019) [16]	**Pop: **87 pediatric patients (age 3 months–18 years) with idiopathic or heritable PAH diagnosed in Japan, 2005–2012 **Group: **iPAH and PAH	**Thres:** PAH defined by RHC: mean PAP ≥25 mmHg at rest, PAWP ≤15 mmHg, and PVR index ≥3 Wood units·m^2^ **Ref: **RHC-confirmed PAH	Retrospective observational	No Reference	Screening group had higher WHO-FC I/II proportion (96% vs. 60%, *p* < 0.001), longer 6MWD (420 ± 109 vs. 327 ± 104 m, *p* < 0.001), lower plasma BNP (149 ± 290 vs. 398 ± 559 pg/mL, *p* = 0.045), and lower IV epoprostenol use at final visit (14% vs. 50%, *p* = 0.004) with similar mean PAP and PVR index	1. Small sample size (n = 87) 2. Potential age bias in screening group 3. Inter-institutional treatment variations 4. Lack of cost-effectiveness analysis 5. Elatively short follow-up period
DuBrock et al. (2024) [19]	**Pop: **39,823 PH-likely and 219,404 control patients from Mayo Clinic; 6045 PH-likely and 24,256 control patients from VUMC for external validation **Group: **PH	**Thres: **mPAP >20 mmHg (RHC) or TRV >3.4 m/s (Echo) **Ref: **RHC or Echocardiography	**Design: **Retrospective **Val: **External (VUMC) & Internal (Random split)	CNN	Model achieved an AUC 0.92 (95% CI: 0.92–0.92) in Mayo and 0.88 (0.87–0.88) in VUMC validation. Early detection (6–18 mo pre-dx) achieved AUC 0.86 (Mayo) / 0.81 (VUMC). Scores correlated with severity; DCA indicated superior utility vs. physician review	1. Reliance on Echo for labels (misclassification risk) 2. Selection bias (referral for Echo/RHC) 3. Retrospective design
Kwon et al. (2020) [20]	**Pop: **Development: n = 24,202 (56,670 ECGs); Internal Val: n = 3174; External Val: n = 10,865 (Hospital-based) **Group:** PH	**Thres:** PASP >35 mmHg or TR gradient >32 mmHg **Ref: **Echocardiography	**Design: **Retrospective **Val: **Internal (Hospital 1) & External (Hospital 2)	Ensemble Neural Network (CNN)	Model achieved an AUC of 0.859 (95% CI: 0.855–0.863) in internal validation and 0.902 (0.900–0.905) in external validation. High-risk patients showed significantly higher PH incidence (31.5% vs. 5.9%, *p* < 0.001). Saliency maps identified P, S, and T waves as key predictive features.	1. Reliance on echocardiography instead of RHC 2. Data derived from only two Korean hospitals 3. Limited model interpretability, functioning as a “black box”
Nemati et al. (2024) [23]	**Pop:** Primary intended-use dataset: 413 symptomatic participants (252 PH-positive and 161 PH-negative) from IDENTIFY-PH and IDENTIFY Group 4. The secondary-threshold analysis included 488 participants after adding 75 subjects with mPAP 21–24 mmHg. The complete model-development cohort included 456 participants (252 PH-positive and 204 PH-negative); 43 additional PH-negative subjects from IDENTIFY Group 3 were used only for model training. A separate, non-overlapping cohort of 161 subjects from RADPH, CADLAD, and IDENTIFY Group 2 was used for feature selection **Group:** Pulmonary hypertension; subgroup analyses included precapillary, isolated postcapillary, and combined precapillary and postcapillary PH	**Thres: **mPAP ≥25 mmHg (primary; 2015 ESC/ERS); mPAP ≥21 mmHg (secondary; the study’s operationalization of the 2022 ESC/ERS definition) **Ref:** iRHC for PH-positive participants; low probability of PH on echocardiography within 90 days, together with absence of diastolic dysfunction, for PH-negative participants	**Design:** Machine-learning model-development study using data from multiple prospective clinical studies, with a separate feature-selection dataset drawn from prospective and retrospective sources **Val:** Internal validation using stratified five-fold out-of-fold cross-validation repeated 100 times; no independent external-validation results were reported in this article	Stacked ensemble formed by averaging Elastic Net and Random Forest predictions	Model achieved an AUC of 0.93 (95% CI: 0.91–0.95) with 87% sensitivity and 83% specificity for the 25 mmHg threshold. Performance remained high (AUC 0.91; 95% CI: 0.88–0.94) using the 21 mmHg threshold. Results were consistent across gender, age, BMI, and PH subtypes. Feature importance highlighted conduction and repolarization metrics	1. Reliance on out-of-fold cross-validation 2. Definition of the non-diseased cohort based on echocardiography instead of RHC 3. Limited dataset size necessitating the use of hand-crafted features rather than end-to-end DL

AI, artificial intelligence; ECG, electrocardiogram; PH, pulmonary hypertension; PAH, pulmonary arterial hypertension; RHC, right heart catheterization; PAP, pulmonary artery pressure; PAWP, pulmonary artery wedge pressure; PVR, pulmonary vascular resistance; WHO-FC, World Health Organization functional class; 6MWD, 6-minute walk distance; BNP, B-type natriuretic peptide; IV, intravenous; VUMC, Vanderbilt University Medical Center; mPAP, mean pulmonary artery pressure; TRV, tricuspid regurgitation velocity; CNN, convolutional neural network; AUC, area under the curve; DCA, decision curve analysis; TR, tricuspid regurgitation; DL, deep learning.

### 3.3 Chest X-Ray and AI Enhancement

While ECG provides a rapid and accessible assessment of electrical and rhythm-related abnormalities associated with PH, its limited sensitivity and specificity in early disease underscore the need for complementary imaging modalities that can capture structural and pulmonary vascular changes. CXR remains the most commonly used radiological examination globally, primarily due to its accessibility, non-invasive nature, and affordability. Despite its widespread use, CXR’s capacity to detect cardiovascular diseases, assess cardiac function, and predict potential cardiovascular events is often underappreciated. This is partly due to its limited diagnostic accuracy and the variability in interpretation among different observers. As a result, more advanced diagnostic methods are typically required to evaluate cardiovascular conditions. Given the rising prevalence of cardiovascular diseases, it is essential to develop rapid, reliable, and easily accessible tests for efficient diagnosis of these common health issues [25,26]. CXR often reveals abnormalities in patients with PH, though a normal result does not rule out the condition. Radiographic indicators of PH typically include changes in the cardiac silhouette, particularly enlargement of the right heart (right atrium [RA] and right ventricle [RV]) and pulmonary artery (PA), occasionally accompanied by peripheral vessel pruning. Additionally, features suggesting the underlying cause of PH, such as LHD or lung conditions, may also be visible [1,27].

#### Automated Image Analysis and Feature Extraction for Disease Prediction, Diagnosis and Classification

AI models have the potential to detect subtle patterns in imaging studies that may be overlooked by human observers, as well as explore correlations between these imaging features and clinical data. As a result, there has been growing interest in leveraging AI to improve diagnostic accuracy in CXR analysis [28]. AI models commonly utilize convolutional neural networks (CNNs) for image analysis, which are highly effective in automating feature extraction from images without depending on human interpretation. This makes CNNs particularly powerful for enhancing diagnostic capabilities [29]. These automated models, including those that analyze imaging patterns, offer a more reliable, efficient, and rapid way to predict clinical conditions, diseases, and outcomes compared to traditional clinician-based interpretations. These models are particularly valuable in clinical workflow with limited access to specialists and can enhance workflow accuracy in high-volume settings. Currently, AI-based studies are focusing on analyzing features from CXR to advance diagnostic capabilities.

Although certain ﬁndings can be suggestive of possible PH in patients undergoing CXR when suspected of the disease, known features of CXR have low sensitivity and speciﬁcity. Considering this, Kusunose et al. [30,31] developed and validated a CNN model using CXR to detect elevated pulmonary artery pressure (PAP) and predict prognosis in patients with suspected PH. In a cohort of 900 patients, the AI model outperformed traditional CXR measurements and human readers in identifying elevated PAP (AUC 0.71 vs. 0.60 and 0.63), and those identified by AI as high risk had twice the incidence of heart failure. In a subsequent study of 142 patients with connective tissue disease, the same AI model helped predict exercise-induced PH (EIPH), with its predictive value enhanced by integrating basic clinical variables (AUC improved from 0.65 to 0.74). These studies highlight the potential of AI-enhanced CXR interpretation in screening and risk stratification for PH, particularly in resource-limited settings.

Zou et al. [32] trained and validated three DL models (ResNet50, Xception, and Inception V3) using chest radiographs and echocardiography-derived pulmonary artery systolic pressure (PASP) values from 762 patients to classify PH-related abnormalities and predict PASP. Their models outperformed manual interpretation by a radiologist in classifying PH presence. Despite promising results, the study faced limitations such as lack of multimodal input, age imbalance between PH-positive and negative groups, and a relatively small dataset. Nonetheless, the approach demonstrates the potential of AI-based CXR analysis as a non-invasive, accessible PH screening tool. Besides, Imai et al. [33] employed the ResNet50 model, pre-trained on ImageNet-1k, for image classification and developed a DL algorithm based on CXR images to predict PAH. The algorithm achieved an impressive area under the receiver operating characteristic curve (AUC) of 0.988. With an AUC threshold of 0.69, the sensitivity and specificity were 0.933 and 0.982, respectively. The algorithm outperformed physicians in detection accuracy. However, limitations such as the small sample size, lack of specificity for different types of PH, variations in population characteristics, and concerns regarding the algorithm’s accuracy and applicability indicate the need for further trials and refinement.

Recent CXR-based AI studies have extended the application of chest radiography from PH detection to multi-class disease classification, motivated by the need for scalable tools to support early phenotyping in routine clinical settings [34,35]. These studies employed deep convolutional neural networks trained on frontal chest radiographs to differentiate PH subtypes using retrospective cohorts, with disease labels derived from a combination of clinical diagnosis and noninvasive imaging criteria rather than uniform hemodynamic confirmation. From a clinical perspective, such models demonstrate the potential of CXR–based AI to function as a front-line triage or pre-classification tool, particularly in environments where access to advanced imaging or RHC is limited. However, the current evidence base is constrained by heterogeneous reference standards, class imbalance, and predominantly internal validation, limiting confidence in subtype-specific clinical decision-making. Consequently, while CXR-based AI classification highlights an important proof of concept, its primary value at present lies in risk stratification and referral support rather than definitive etiologic classification, underscoring the need for standardized definitions, external validation, and integration within multimodal diagnostic pathways.

Despite outperforming clinicians to some extent in PH detection, these models still face important limitations in clinical applicability and generalizability. Lack of multimodal input, limited sample diversity, inability to distinguish PH subtypes, and need for external validation. These findings underscore the promise of CXR-based AI models in PH screening, especially in resource-limited settings, while highlighting the need for larger, more robust studies to confirm generalizability and clinical integration. Table 2 (Ref. [22,26,30,31,32,33,34,35]) summarizes the key findings related to the application of AI in CXR as discussed in this section.

**Table 2.** **Summary of AI in CXR applications in PH**.

Author (Year)	Population & PH-group	Threshold & Reference standard	Study design & Validation	Model	Key findings & Performance	Limitations
Liu et al. (2025) [22]	**Pop:** Development: 42,299 patients (Hospital A); Int Val: 25,512 patients; Ext Val: 16,736 patients (Hospital B). Group: Elevated Pulmonary Arterial Pressure (ePAP). **Group: **ePAP	**Thres:** PASP >50 mmHg (TRV >3.4 m/s) **Ref:** Echocardiography	**Design:** Retrospective Multimodal DLM Development **Val:** Internal (Hospital A) & External (Hospital B)	CNN (ECG) + DenseNet-121 (CXR) + XGBoost (Fusion)	Combined ECG+CXR model achieved AUCs of 0.8644 (internal) and 0.8734 (external), outperforming single modalities. It showed high NPV (>98%). Positive predictions were associated with significantly higher cardiovascular mortality (HR 6.08, 95% CI: 4.66–7.95) and new-onset left ventricular dysfunction	1. Reliance on echocardiography, limiting definitive hemodynamic diagnosis 2. Exclusively Taiwanese study population, requiring validation in other racial groups for generalizability 3. Limited clinical interpretability due to the “black box” nature of DL models
Ueda et al. (2023) [26]	**Pop: **Train: 12,897; Int Val: 1432 (3 sites); Ext Val: 2617 (Site D). **Group:** Cardiac Function & Valvular Heart Disease	**Thres:** LVEF (40%, 50%), TRV (2.8, 3.4 m/s), Valvular (Mod-Severe). **Ref: **Echocardiography	Design: Multi-institutional Retrospective Development. **Val:** Internal (5-fold CV) & External (Dataset D)	EfficientNet (Multilabel DL)	External validation achieved AUC 0.92 (95% CI: 0.90–0.95) for LVEF <40% and 0.85 (0.83–0.87) for TRV >2.8 m/s. Valvular disease detection was robust (AUCs 0.83–0.92). Saliency maps confirmed focus on cardiac structures	1. Reliance on echocardiography as the reference standard, whereas cardiovascular MRI may provide more accurate assessment 2. Model trained exclusively on standing posteroanterior radiographs
Kusunose et al. (2020) [30]	**Pop:** Train/Val: 900 retrospective patients (10-fold CV); Temporal Val: 55 prospective patients. **Group: **Suspected PH	**Thres: **mPAP >20 mmHg **Ref: **RHC	**Design:** Retrospective (Train) & Prospective (Val) **Val:** Independent Temporal Validation (n = 55)	CNN	AI achieved AUC 0.71 (0.63–0.78), surpassing human readers (0.63). AI-predicted PH was associated with significantly higher heart failure admission risk (HR 2.12, 95% CI: 1.18–3.96). The model demonstrated limited performance when used alone but provided prognostic value	1. Relatively modest sample size for DL, potentially limiting model robustness 2. Inability to differentiate PH subtypes (e.g., pre- vs. postcapillary) due to insufficient data 3. Single-center study design, limiting generalizability
Kusunose et al. (2022) [31]	**Pop: **n = 142 patients with SSc/MCTD undergoing 6-min walk stress echocardiography (52 EIPH, 90 non-EIPH). **Group:** Exercise-Induced Pulmonary Hypertension (EIPH)	**Thres:** ΔmPAP/ΔCO >3.3 mmHg/L/min and ex-mPAP >25 mmHg **Ref: **6-min Walk Stress Echocardiography	**Design: **Retrospective Cohort **Val:** Clinical Cohort (SSc/MCTD)	CNN	The DL model significantly improved the prediction of EIPH when added to clinical variables (AUC increased from 0.65 to 0.74, *p* = 0.046). AI probability scores were significantly higher in the EIPH group compared to non-EIPH. High-risk patients identified by AI may benefit from early stress echocardiography assessment.	1. Reliance on stress echocardiography instead of RHC 2. Small sample size with significant gender bias 3. Inconsistent availability of key biomarkers, such as natriuretic peptides
Zou et al. (2020) [32]	**Pop: **Train: 641 (561 pre-train, 80 val); Int Test: 80; Ext Test: 41. Total n = 762 from 3 Chinese institutes. **Group: **PH	**Thres:** PASP ≥40 mmHg **Ref:** Echocardiography	**Design:** Retrospective Multicenter Study. **Val:** Internal (8-fold CV) & External (Dataset from 3rd institute)	CNN (Inception V3, ResNet50, Xception)	Inception V3 achieved the best performance with an AUC of 0.970 in the internal test and 0.967 in the external test. For continuous PASP prediction (regression), the model achieved a mean absolute error (MAE) of 7.45 mmHg (internal) and 9.95 mmHg (external)	1. Reliance on echocardiography (PASP) instead of gold-standard RHC, with potential measurement error 2. Use of frontal chest radiographs only, excluding lateral views and clinical variables 3. Limited number of severe PH cases (>80 mmHg), potentially biasing regression performance 4. Significant age differences between PH and control groups
Imai et al. (2024) [33]	**Pop: **Train: 418 images; Test: 101 images (Total 519 CXRs from 145 PAH patients, 260 Controls). **Group:** PAH.	**Thres:** Clinical Diagnosis of PAH **Ref:** RHC/Expert Consensus	**Design:** Retrospective Single-center Study. **Val:** Internal Testing (101 images)	ResNet50 (Pre-trained on ImageNet).	Algorithm achieved an AUC of 0.988, with 93.3% sensitivity and 98.2% specificity, outperforming board-certified respirologists. Grad-CAM analysis indicated the model primarily focused on pericardial areas rather than just vascular pruning	1. Relatively small sample size due to the rarity of PAH, limiting model robustness 2. Single-center, retrospective study design 3. Model trained specifically on PAH, with uncertain applicability to other PH subtypes
White et al., (2025) [34]	**Pop:** n = 1,682 subjects (750 healthy/asymptomatic) excluding systolic dysfunction/confounders. **Group:** Pulmonary Venous Hypertension (PVH) / LVDD	**Thres: **PVH Stages / LVDD Grades (0–4). **Ref: **Echocardiography	**Design: **Retrospective Data-Mining. **Val:** Internal (Train/Val/Test Split)	Transformer-based AI Model (PVPI) / ResNet-50 (Segmentation)	PVPI model achieved overall accuracy of 0.89 and balanced accuracy of 0.86 for PVH detection. It showed high normalized class accuracies (Normal 0.91, PVH 0.90), suggesting AI-assisted staging may exceed human sensitivity for identifying early LVDD progression	1. Relatively small sample size per subgroup due to strict exclusion criteria, potentially limiting real-world generalizability 2. Need for further validation of AI model performance in complex cases with anatomical or physiological comorbidities
Kıvrak et al. (2023) [35]	**Pop: **6642 CXR images (5 PH subgroups + Controls) retrospectively collected from multiple centers. **Group:** PH	**Thres: **Clinical Diagnosis (PH types) **Ref: **Multimodality Assessment (Echo/RHC)	**Design: **Retrospective Multicenter Classification **Val:** Internal (Cross-validation)	EfficientNetb0 (Patch-based Deep Feature Extraction)	Model achieved 86.14% overall accuracy and average AUC 0.945 across six categories (5 PH types vs. Control). The patch-based EfficientNetb0 approach successfully differentiated complex PH etiologies from normal controls	1. Inability to perform RHC on healthy controls, relying on clinical assessment and potentially affecting label precision 2. Need for prospective validation to assess clinical utility in screening undifferentiated dyspnea populations

ePAP, elevated pulmonary artery pressure; PASP, pulmonary artery systolic pressure; DLM, deep learning model; CXR, chest X-ray; CV, cross-validation; SSc/MCTD, systemic sclerosis/mixed connective tissue disease; LVDD, left ventricular diastolic dysfunction.

### 3.4 Echocardiography and AI Integration

Although CXR can reveal indirect signs of PH, such as pulmonary artery enlargement or right ventricular prominence, its qualitative nature and low diagnostic accuracy necessitate more functional and hemodynamic assessments, for which echocardiography remains the primary noninvasive tool. Echocardiography plays a crucial role in diagnosing and managing cardiac conditions. It is one of the few imaging techniques that provide real-time visualization and can identify a wide range of abnormalities. Accurate assessment of cardiac structure and function through echocardiography is essential for clinical diagnosis and treatment planning [36]. Echocardiography is widely used for PH screening [1].

We focus on the diagnosis in PH through Echocardiography. Echocardiography is a key noninvasive tool for assessing right ventricular pressure overload and dysfunction in PH, providing detailed evaluation of cardiac structure, ventricular function, valvular disease, and hemodynamic parameters, and helping identify etiologies such as left heart disease or congenital heart disease. However, despite its clinical value, echocardiography cannot definitively diagnose PH, which requires confirmation by RHC [1]. Besides, even in the development of new technologies (3-dimensional echocardiography, speckle-tracking, semi-automated analysis, etc.), the final analysis is strongly dependent on operator experience [37]. The Echocardiography interpretation in clinical practice is not intuitionistic as ECG and DR. The results require extensive expertise and training, posing challenges for its widespread use in primary care and rural settings. What is more, this also includes subjective interpretation of data points, resulting in poor inter and intra-observer correlation and a reliance on operator dependant acquisition of images and key measurements that dictate results [38]. AI has the potential to enhance echocardiography-based PH diagnosis by reducing human error, identifying subtle or hidden features, and quantifying disease-specific patterns. ML models can learn intrinsic echocardiographic features, improving diagnostic precision and minimizing inter- and intra-operator variability. When combined with clinical interpretation, AI can boost accuracy and broaden access to expert-level evaluation, especially in settings lacking specialized expertise [39].

#### 3.4.1 Automatic Structure Recognition, Segmentation and Quantification

Zhang et al. [40] trained and evaluated CNN network models for multiple tasks in echocardiography including image classification of 23 standard views and segmentation. This ten years study used 14,035 echocardiograms to train the models that had the capacity for interpretation and diagnosis of pathology, a truly comprehensive application of AI in echocardiography. They established a pipeline for echocardiogram interpretation, including (1) view identification, (2) image segmentation, (3) quantification of structure and function, and (4) disease detection. This is an integrated and progressive research, which enlightens many cardiologists to see a potential role for AI in the echocardiography. Kusunose et al. [37] summed up utilization of AI in Echocardiography from four aspects, including AI for View Classifications AI for Size and Function AI for Wall Motion Abnormality and AI for Diagnosis of Cardiovascular Diseases. Kusunose et al. [38] employed a CNN to enhance the detection of regional wall motion abnormalities. Their findings revealed an AUC similar to that of cardiologists and sonographers (0.99 vs. 0.98, respectively; *p* = 0.15), but a notably higher AUC compared to junior medical doctors (0.97 vs. 0.83, respectively; *p* = 0.003). It is evident that AI plays a multifaceted role in the field of echocardiography, with applications spanning from basic image analysis to complex diagnostic processes. This integration unfolds progressively, starting from view determination, followed by image segmentation, structural and functional quantification, and ultimately applying in disease diagnosis.

#### 3.4.2 Disease Detection

Anand et al. [41] conducted a retrospective study of adults who underwent RHC at the Mayo Clinic between January 1, 2012, and December 31, 2019, with a transthoracic echocardiogram within one week. The study included 7,853 patients, divided into training (n = 5024), validation (n = 1275), and test sets (n = 1554). A gradient boosting machine (GBM) with grid search optimization was used to predict PH, defined by RHC (mean pulmonary artery pressure >20 mmHg). The model achieved an AUC of 0.83 (95% CI, 0.80–0.85) on the test set, with 82% accuracy, 88% sensitivity, 89% positive predictive value, and 54% negative predictive value. The model was robust even when tricuspid regurgitation (TR) velocity could not be measured (AUC = 0.785). Key findings included that 81% of patients had PH, and 68% had abnormal right ventricle size. The study concluded that ML using clinical and echocardiographic data can predict PH without relying on TR velocity. This approach may help identify patients with a low likelihood of PH, potentially reducing unnecessary RHC. However, further research is needed to improve specificity and negative predictive value. The model was designed to handle missing data without imputation, improving real-world applicability. Feature selection focused on measurable characteristics in most cases, and descriptive features were standardized for better model stability. However, the retrospective design may introduce bias, and some clinical data were missing. Although the model showed high sensitivity, its specificity and negative predictive value were suboptimal, limiting its standalone clinical use. Furthermore, 80% of patients had higher-than-normal pulmonary artery pressures, which may affect the model’s generalizability and risk of overfitting, despite efforts to mitigate this with cross-validation.

Sun et al. [42] developed a Chamber Attention Network (CAN) model for PAH, comprising three main components: (i) a CNN-based view classifier to select diagnostically relevant echocardiographic views (A4C and PLAX), (ii) U-Net–based segmentation of cardiac regions and chambers, and (iii) a Grad-CAM–guided chamber attention module that quantifies chamber importance and reconstructs images for final classification using ResNet50; results from multiple images and views were combined using a voting strategy to enhance reliability. Clinically, this framework is positioned as an AI-assisted diagnostic tool for PAH screening/identification from routine echocardiography, with particular relevance for telemedicine-supported workflows. Key findings showed that CAN achieved high internal validation performance and retained generalization on an external hospital dataset, while its attention vectors aligned with clinical knowledge (dominant emphasis on the right ventricle) and additionally suggested potential diagnostic contribution from other chambers, thereby improving transparency and trustworthiness for clinical implementation.

#### 3.4.3 Classification and Risk Stratification

Hirata et al. [43] developed and evaluated a model using ML algorithms, including logistic regression, support vector machines, random forests, and XGBoost classifiers, based on 24 clinical variables (age, gender, height, weight, and echocardiographic data). A stratified 10-fold cross-validation was used, and Shapley Additive exPlanations (SHAP) analysis helped interpret the model. The study analyzed two independent cohorts (720 patients in the derivation cohort and 165 in the validation cohort), with patients classified based on RHC into non- PH, precapillary PH, and postcapillary PH groups. The models were compared to guideline-based echocardiographic assessments. The results showed that the logistic regression classifier had the highest accuracy, with AUC for non-PH, precapillary PH, and postcapillary PH groups of 0.789, 0.766, and 0.742, respectively. In the derivation cohort, the ML model outperformed guideline-based assessments, while in the validation cohort, the accuracy was similar to the guideline-based method. SHAP analysis identified key predictors, including tricuspid regurgitation velocity (TRV), heart rate (HR), and weight, while echocardiographic factors like left atrial volume index (LAVi) and E/A ratio were important for predicting postcapillary PH. This study shows that ML can aid in classifying PH using echocardiographic data, offering quantitative results and improving reproducibility. It highlights the importance of echocardiographic markers and clinical features (like HR and weight) for accurate PH classification and a better understanding of PH’s pathophysiology. Table 3 (Ref. [40,41,42,43,44]) summarizes the key findings related to the application of AI in Echo as discussed in this section.

**Table 3.** **Summary of AI in Echo applications in PH**.

Author (Year)	Population & PH-group	Threshold & Reference standard	Study design & Validation	Model	Key findings & Performance	Limitations
Zhang et al. (2018) [40]	**Pop: **Train/Eval: 14,035 echocardiograms (10-year period); Clinical **Val: **8666 studies. Group: PAH	**Thres:** Disease Probability (Continuous). **Ref: **Clinical Diagnosis (Medication confirmed for PAH) / Manual Segmentation	**Design: **Retrospective Cohort (Automated Pipeline Development). **Val: **5-fold Cross-validation & Clinical Workflow Comparison	CNN (13-layer View Classification) & U-Net (Segmentation)	Automated pipeline successfully identified views (96% accuracy) and segmented chambers. Disease detection models achieved AUCs of 0.93 (HCM), 0.87 (Amyloid), and 0.85 (PAH). Automated metrics showed high agreement with manual measurements and commercial software	1. Lack of differentiation between specific hypertrophic disease subtypes, limited to binary detection 2. Absence of comparison with models using hand-selected clinical features
Anand et al. (2024) [41]	**Pop:** 7853 patients with paired RHC and TTE were split into training (n = 5024), validation (n = 1275), and testing (n = 1554) sets. **Group:** PH	**Thres: **mPAP >20 mmHg **Ref:** RHC	**Design:** Retrospective Cohort **Val:** Internal (Train/Val/Test Split)	Gradient Boosting Machine (XGBoost)	The model achieved an AUC of 0.83 (95% CI: 0.80–0.85) with 88% sensitivity and 82% accuracy in the testing set. While positive predictive value was high (89%), the negative predictive value was limited (54%) due to the cohort’s high PH prevalence	1. High PH prevalence (81%) due to catheterization-based recruitment, limiting applicability to lower-risk screening populations 2. Notable performance decline from training to testing, suggesting potential overfitting
Sun et al. (2024) [42]	**Pop: Int:** 13,912 subjects (2122 PAH); **Ext:** 3150 subjects (High-altitude cohort from Tibet). **Group:** PAH	**Thres:** mPAP ≥25 mmHg (estimated via Echo). **Ref:** Clinical Echocardiography Diagnosis	**Design:** Retrospective Multicenter Study. **Val:** Internal (Train/Val) & External (Independent Hospital)	Chamber Attention Network (CAN) [ResNet50 backbone]	CAN achieved AUCs of 0.979 (internal) and 0.862 (external) for A4C views, outperforming ResNet50 (AUC 0.665). Integrating A4C and PLAX views yielded 97.6% PPV in external testing. Saliency maps confirmed accurate focus on right heart chambers	1. Inability to predict disease severity or progression due to dataset constraints 2. External validation cohort drawn exclusively from a high-altitude region (Tibet), introducing potential physiological confounders
Hirata et al. (2024) [43]	**Pop: **Derivation: n = 720; Prospective Val: n = 165. Total 885 patients undergoing RHC. **Group:** Non-PH, Precapillary PH, Postcapillary PH	Thres: Pre-cap PH (mPAP >20, PAWP ≤15); Post-cap PH (PAWP >15). **Ref: **RHC	**Design:** Retrospective Derivation & Prospective Validation. **Val:** Prospective Cohort (n = 165)	Logistic Regression (Elastic Net) [Best Performer vs. XGBoost/RF].	The Elastic Net model achieved AUCs of 0.789 (Non-PH), 0.766 (Precapillary), and 0.742 (Postcapillary), outperforming guideline-based assessment (accuracy 57% vs. 47%). It effectively stratified patients using standard echo parameters like TR velocity and E/e’ ratio	1. Exclusion of patients with atrial fibrillation, limiting generalizability to this common subgroup 2. Reliance on standard parameters without incorporation of advanced right ventricular metrics (e.g., TAPSE or strain) due to inconsistent availability
Fortmeier et al. [44]	**Pop: Dev:** n = 116 (from 162 total); **Ext Val:** n = 142. All patients underwent TTVI for severe TR. **Group:** Severe Tricuspid Regurgitation (TR) & PH	**Thres:** Predicted mPAP >29.9 mmHg (Mortality Risk). **Ref:** RHC	**Design:** Retrospective Multicenter Derivation. **Val:** External Validation (Independent Institution)	Extreme Gradient Boosting (XGBoost) [Decision Trees].	XGBoost corrected echocardiographic underestimation of PH, reclassifying patients accurately. Predicted mPAP >29.9 mmHg was significantly associated with increased 2-year all-cause mortality (HR 2.4, 95% CI: 1.1–5.2) in internal testing and confirmed externally (HR 2.9, 95% CI: 1.5–5.7)	1. Reliance on expert-derived echocardiographic parameters rather than raw images, introducing inter-observer variability 2. Exclusive focus on a severe tricuspid regurgitation population undergoing intervention, limiting generalizability to milder or untreated cases

HCM, hypertrophic cardiomyopathy; TTE, transthoracic echocardiography; RF, random forest; A4C, apical four-chamber view; PPV, positive predictive value; PLAX, parasternal long-axis view; TTVI, transcatheter tricuspid valve intervention.

Fortmeier et al. [44] addressed a clinically important diagnostic gap in echocardiography—namely, the systematic underestimation of PH severity in patients with severe TR, which can obscure risk assessment before transcatheter tricuspid valve intervention (TTVI). The objective was to improve noninvasive PH assessment by predicting mPAP using echocardiographic parameters alone, with RHC as the gold-standard reference. Using a bicenter registry (2016–2020), the authors trained an extreme gradient boosting (XGB) model in a derivation cohort of patients who had both preprocedural echocardiography and RHC data, and performed external validation in an independent institutional cohort. Clinically, the model functions as an AI-enhanced echocardiographic decision-support tool to refine PH assessment and risk stratification in high-risk TR populations undergoing TTVI. Key findings showed that conventional echocardiographic sPAP significantly underestimated invasively measured pressures, whereas the XGB model—using nine routine echo variables—could reliably predict mPAP and identify a high-risk subgroup with significantly worse 2-year survival after TTVI; this prognostic association was confirmed externally. Limitations include the retrospective design, missing RHC in a subset of eligible patients (selection bias), lack of a centralized echo core lab, and potential measurement variability for volume-sensitive echo parameters (e.g., IVC diameter), which may affect generalizability across centers and clinical states.

### 3.5 Cardiac Magnetic Resonance Imaging Applications

Despite its central role in screening and risk stratification, echocardiography is limited by operator dependency and acoustic window constraints, highlighting the value of CMR imaging as a reproducible modality for comprehensive evaluation of right ventricular structure, function, and tissue characteristics. CMR provides accurate and reproducible assessments of atrial and ventricular size, morphology, and function. Through the application of tagging techniques or post-processing feature tracking, it can further provide information on myocardial strain in the right ventricle/left ventricle (RV/LV). CMR is also capable of measuring blood flow in the PA, aorta, and vena cava, allowing for the quantification of stroke volume (SV), intracardiac shunts, and retrograde blood flow. By combining contrast-enhanced MR angiography, late gadolinium enhancement imaging of the myocardium, and pulmonary perfusion imaging, a comprehensive evaluation of the heart and pulmonary vasculature can be achieved. However, its limitation lies in the lack of a standardized method for estimating PAP. Despite the cost and availability constraints that limit its use in the early diagnosis of PAH, CMR demonstrates high sensitivity in detecting early signs of PAH [1].

CMR enables non-invasive evaluation of the heart and associated vascular structures. Beyond detecting structural abnormalities such as myocardial scars, CMR also offers quantitative data on myocardial volume and provides estimates of physiological function, including myocardial performance [45]. CMR requires time and cost-intensive expert assessment. However, AI has the potential to address these challenges, and efforts to improve the efficiency of CMR analysis have primarily focused on the automatic segmentation of cardiac structures [46]. AI technologies for automated diagnosis and prognosis are continuously emerging. These AI methods can rapidly generate clinical indicators and demonstrate accuracy comparable to that of expert human clinicians. Looking ahead, AI is likely to play a significant role in enhancing the efficiency of CMR reporting and improving prognosis through risk stratification in cardiovascular disease patients.

#### 3.5.1 Automated Structural Recognition and Quantitative Analysis, Functional Assessment, and Image Enhancement

In a 2017 study, Dawes et al. [47] explored the application of high-resolution cardiac imaging data, particularly from CMR, in ML models. By manually marking six key anatomical points on CMR images, they constructed a reference atlas specifically for PH-affected hearts. This enabled the automated generation of 30,000 data points per patient from subsequent imaging scans, capturing the motion of the heart from end-diastole to end-systole. A receiver operating characteristic (ROC) analysis for one-year survival was then performed. Using supervised principal component analysis, a three-dimensional motion score was derived, which significantly outperformed traditional CMR measurements, hemodynamic data, and exercise capacity in predicting the outcomes of 256 PH patients (AUROC: 0.73 vs. 0.60; *p* < 0.001). This innovative approach combined cardiac imaging with ML, providing valuable insights for survival prediction in PH patients through advanced functional modeling techniques.

Additionally, the atlas-based segmentation approach maintained anatomical correspondence of the three-dimensional mesh between patients, improving the accuracy and reproducibility of cardiac structure and function analysis. This approach contributed to a deeper understanding of how overall cardiac function affects survival mechanisms. Building on the previous research, Attard et al. [48] performed 3D modeling of the RV in PH patients, analyzing the relationships between its phenotype, hemodynamics, metabolite levels, and wall stress, and evaluating the predictive role of wall stress on survival. Their findings revealed that RV wall stress, a significant prognostic factor, can be calculated and is also associated with plasma levels of key prognostic metabolites, such as N2, N2-dimethyl-guanosine. These advancements highlighted a novel connection in the pathobiology-phenotype relationship of PAH, linking maladaptive alterations in RV geometry with metabolic stress, especially in the context of increased clinical risk. Their research developed an automated method for 3D modeling of the RV in PH patients, integrating quantitative analysis of afterload, biomarkers, and structural adaptation relationships. For the first time, they conducted 3D mapping of wall stress in PH patients, revealing regional variations and their relationships with myocardial hypertrophy, remodeling, and ejection fraction. They also found associations between various metabolites and RV function and wall stress, providing insights into the mechanisms of RV adaptation.

Duan et al. [49] relying on the Simultaneous Segmentation and Landmark Localization Network (SSLLN) and the introduction of anatomical shape priors, data preprocessing, and data augmentation, processed and analyzed CMR images from 1831 healthy subjects and 649 PH patients. An automated cardiac image dual-ventricle segmentation pipeline was proposed, capable of handling both low-resolution (LR) and high-resolution (HR) CMR data. Accurate, high-resolution, and anatomically smooth 3D models of the dual ventricles were generated effectively in both healthy and pathological conditions. This study is significant in CMR anatomy, as it clearly introduces anatomical shape prior knowledge and uses atlas propagation to effectively overcome the effects of image artifacts and pathological morphological changes, thereby improving segmentation quality.

#### 3.5.2 Screening and Diagnosis

Swift et al. [50] used ML methods to extract disease features from CMR for the diagnosis of PAH performed. They preprocessed of CMR images, including manually marking landmark points for registration and generating an elliptical mask to focus on the anatomical region of interest in the whole area. They also scaled the image resolution to reduce overfitting. They then applied a tensor-based ML method—Multilinear Subspace Learning (MPCA)—to reduce the dimensionality of the CMR image tensor samples. Fisher’s discriminant ratio was used to select features, and the final classification model was learned using Support Vector Machine (SVM) or Logistic Regression (LR) classifiers. They identified 220 patients from the ASPIRE registry who had either untreated PAH or no evidence of PH, with CMR and RHC performed within 48 hours (150 PAH patients and 70 without PH). They extracted, visualized, and interpreted diagnostic features associated with PAH and compared the diagnostic accuracy with standard CMR measurement methods. The conclusions were as follows: (1) The model could identify diagnostic features without manual image segmentation, reducing measurement discrepancies caused by human factors. The three-point manual marking method was simple, fast, and easy to operate. (2) The model was capable of comprehensively identifying potential diagnostic features from the images, including both ventricular and extra-ventricular regions, which helped uncover new diagnostic information. (3) It utilized a relatively simple and interpretable linear method, making it easy to directly visualize feature importance, aiding in result interpretation. The tensor-based ML method they developed for CMR showed high accuracy in diagnosing PAH. The newly learned features demonstrated diagnostic potential, slightly outperforming traditional manual measurement indicators while being more cost-effective and less variable. Additionally, they identified new features, such as those from short-axis imaging, four-chamber heart analysis, and right ventricle blood pool characteristics, while confirming the diagnostic value of some known features.

Wang et al. [51] developed a video-based Swin Transformer (VST) model for diagnosing 11 types of cardiovascular diseases (CVDs), including PAH, using a large, representative CMR dataset of 9,719 individuals. The model includes a two-stage approach: (1) a screening model using cine CMR (SAX and 4CH views) to detect abnormalities, and (2) a diagnostic model combining cine and late gadolinium enhancement (LGE) images for precise disease classification. Compared to traditional CNN-LSTM models, the VST model showed superior performance due to its ability to integrate spatiotemporal information via global self-attention. Comprehensive validation was conducted across internal and external datasets, using multiple preprocessing, augmentation, and interpretability techniques (e.g., Grad-CAM, Shapley values). Results demonstrated that both screening and diagnostic models achieved high accuracy, with the cine-based screening model offering a fast, non-invasive tool for initial detection, and the combined cine+LGE approach significantly enhancing diagnostic accuracy. Notably, the model showed strong performance in detecting PAH, including CMR-negative cases. The AI model also outperformed physicians with varying experience levels. While promising, further optimization and clinical validation are needed to address limitations in model performance and generalizability. Overall, this study highlights the potential of VST-based AI models to improve diagnostic precision and efficiency in CVD, particularly in PAH detection. Table 4 (Ref. [47,48,49,50,51,52,53]) summarizes the key findings related to the application of AI in CMR as discussed in this section.

**Table 4.** **Summary of AI in CMR applications in PH**.

Author (Year)	Population & PH-group	Threshold & Reference standard	Study design & Validation	Model	Key findings & Performance	Limitations
Dawes et al. (2017) [47]	**Pop: **256 patients with newly diagnosed PH (143 women, mean age 63) from a continuous prospective program. **Group:** PH	**Thres:** mPAP ≥25 mmHg **Ref:** RHC	**Design: **Prospective Cohort (Prognostic Modelling) **Val: **Internal (8-fold Cross-Validation)	Supervised ML (3D Cardiac Motion Patterns via PCA)	The ML survival model using 3D cardiac motion significantly improved survival prediction compared to conventional imaging and hemodynamic markers (AUC 0.73 vs. 0.60, *p *< 0.001). It effectively stratified patients by survival time (13.8 vs. 10.7 years for low vs. high risk)	1. Pragmatic grouping of heterogeneous non-congenital PH etiologies, potentially obscuring subgroup-specific patterns 2. Use of all-cause mortality as the sole endpoint, potentially missing cardiac-specific outcomes 3. Reliance on semi-automated segmentation, introducing additional processing time and potential inter-observer variability
Attard et al. (2019) [48]	**Pop:** 312 PH patients, of which 182 had metabolomics data. **Group:** PH	**Thres:** mPAP >25 mmHg **Ref:** RHC	**Design:** Prospective Observational Cohort **Val:** Internal (Statistical Parametric Mapping)	3D Biomechanical Modelling (Mass-univariate Regression)	RV wall stress was an independent predictor of all-cause mortality (HR 1.27, ***p*** = 0.001). Circulating metabolites, specifically DHEA-S (***β ***= 0.31) and N-acetylmethionine, were significantly associated with regional RV wall stress, linking metabolic dysregulation to mechanical maladaptation	1. Observational study design preventing causal inference between metabolites and right ventricular dysfunction 2. Lack of direct tissue or transvenous sampling to confirm cardiac origin of metabolites 3. Significant time interval between CMR and RHC in some patients, potentially affecting hemodynamic correlations
Duan et al. (2019) [49]	**Pop: Dataset 1:** 1831 healthy subjects (UK Digital Heart Project); **Dataset 2:** 649 PH patients (National Centre) with artifact-prone scans. **Group:** Bi-ventricular Segmentation.	**Thres:** N/A (Segmentation Task) **Ref: **Manual Ground Truth Segmentation	**Design:** Algorithm Development & Validation **Val:** Internal Split (Train/Test) & Cross-dataset Evaluation	Shape-Refined Multi-Task DL (FCN + Atlas Propagation)	The proposed shape-refined FCN (SSLLN) generated anatomically smooth 3D bi-ventricular models, effectively overcoming inter-slice shift artifacts common in PH patients where standard methods failed. It achieved high consistency with manual segmentation and outperformed 2D-FCNs, though RV wall segmentation remained challenging	1. Two-stage pipeline, with a computationally expensive shape refinement step 2. Relatively lower segmentation accuracy for the thin right ventricular wall (RVW) 3. Dependence on a pre-constructed atlas, which may bias results
Swift et al. (2021) [50]	**Pop: **220 consecutive patients (150 PAH, 70 no PH) from the ASPIRE registry with paired RHC/CMR. **Group: **PAH	**Thres:** mPAP >25 mmHg, PAWP ≤15 mmHg **Ref:** RHC	**Design: **Retrospective Registry Analysis **Val: **Internal (10-fold Cross-validation)	Tensor-based ML (Multilinear Subspace Learning + SVM/LR)	The tensor-based approach achieved an AUC of 0.92, comparable to or slightly better than standard manual CMR metrics (AUC ~0.91) but significantly faster (<10 s). Feature visualization confirmed discriminative patterns like septal bowing without manual segmentation	1. Reliance on manual landmark placement for image registration, preventing full automation 2. Use of selected 2D cine slices (4-chamber and short-axis) rather than full volumetric data 3. Internal validation via cross-validation only
Wang et al. (2024) [51]	**Pop: **Train: 7900 (Primary); Ext Test: Data from other centers. Total 9719 patients with 11 CVD types (including PAH). **Group: **11 Cardiovascular Diseases (Screening & Diagnosis)	**Thres: **Expert Consensus / Guidelines (11 CVD types). **Ref:** CMR Interpretation.	**Design:** Retrospective Multi-center Development. **Val: **Internal (3-fold CV) & External (Multiple Centers)	Video Swin Transformer (VST) [Two-stage: Screening + Diagnosis]	The two-stage AI model achieved high performance with AUCs of 0.988 (screening) and 0.991 (diagnosis). It outperformed cardiologists in diagnosing specific conditions like PAH and demonstrated robust generalization across internal and external datasets	1. Retrospective study design, requiring prospective validation 2. Cohort limited to Eastern Asian populations, restricting ethnic generalizability 3. Limited number of healthy controls, potentially biasing screening performance
Zeng et al. (2025) [52]	**Pop: **n = 120 patients (77 PH, 43 non-PH) with RHC and CMR data from a single center. **Group:** PH (mPAP Prediction)	**Thres:** mPAP >20 mmHg (Diagnosis). **Ref:** RHC	**Design:** Retrospective Observational Study. **Val: **Internal (10-fold Cross-validation)	XGBoost (Ensemble Learning) & Symbolic Regression (White-box)	Integrated AI model predicted mPAP with high accuracy (R^2^ = 0.992, MAPE = 4.49%) using non-invasive clinical and CMR features. Symbolic regression offered a transparent, interpretable formula for clinical application. The approach effectively identified key biomarkers like wall shear stress	1. Small sample size (n = 120) and single-center retrospective study design 2. Exclusion of patients with incomplete data (n = 37), introducing potential selection bias 3. Need for broader external validation
Alabed et al. (2022) [53]	**Pop:** Train: 539 (Multicenter); Ext Test: 40 multivendor studies (32 centers); Prognostic: 3487 patients (ASPIRE registry). **Group:** Suspected PH & Cardiopulmonary Disease	Thres: Mortality Risk/Hemodynamic Correlation. Ref: RHC & Manual Segmentation	Design: Retrospective Multicenter Development & Prospective Repeatability Study. Val: External (Multivendor) & Clinical Outcome	ResUNet (16-layer Residual Convolutional Neural Network)	AI measurements correlated better with RHC than manual metrics (LVSV γ = 0.72 vs. 0.68) and showed high repeatability (ICC 0.79–0.99). Automated RV mass and ejection fraction significantly predicted mortality in PAH (HR 1.15 and 0.76, *p* = 0.001)	1. Single center (ASPIRE) Validation 2. Direct comparison between AI-derived and manual measurements for mortality limited by differences in trabeculation handling 3. Lack of publicly available and well-documented DL code, restricting independent external reproducibility

PCA, principal component analysis; CMR, cardiac magnetic resonance; ML, machine learning; SVM/LR, Support Vector Machine/Logistic Regression; CVD, cardiovascular disease; MAPE, mean absolute percentage error; ICC, intraclass correlation coefficient; ASPIRE, Assessing the Spectrum of Pulmonary Hypertension Identified at a Referral Centre; REVEAL, Registry to Evaluate Early and Long-term Pulmonary Arterial Hypertension Disease Management.

#### 3.5.3 Classification and Prognostic

Zeng et al. [52] developed an ML model integrating multivariate CMR data to predict PAP in PH patients, aiming to reduce reliance on invasive RHC. The model incorporated key CMR parameters, including right ventricular end-diastolic volume and strain, to predict mPAP. Training and validation were conducted on a dataset of 120 PH patients, with the model achieving an AUC of 0.87 in the internal cohort and 0.82 in the external cohort. Importantly, the model performed well across various PH subtypes, with an AUC of 0.89 for precapillary PH, 0.81for postcapillary PH, and 0.85 for combined subgroups. The study demonstrated that CMR-driven AI can capture detailed cardiac remodeling patterns linked to PH, surpassing single-parameter models in predictive accuracy. However, the study’s retrospective design, use of a single-center dataset, and the lack of prospective external validation represent limitations. Calibration and decision-analytic reporting were also not included, warranting future prospective studies to further assess its clinical utility.

Two studies leveraged ML on CMR to predict PAH and its prognosis. Alabed et al. [53] developed a DL model using multilinear principal component analysis (MPCA) to extract prognostic cardiac features from CMR and predict 1-year mortality in PAH patients. The model demonstrated an AUC of 0.76 for mortality prediction, improving upon traditional risk scores like REVEAL. This model is particularly valuable for identifying high-risk patients in the early stages of PAH. However, it was limited by its retrospective design and reliance on a single-center cohort, requiring further validation. In contrast, Alabed et al. [53] aimed to develop automated AI CMR quantification and validated its utility in a large cohort of 3487 patients, including 920 with PAH. The model correlated strongly with RHC parameters and proved to have a significant prognostic value for mortality prediction in PAH, showing a c-index of 0.83 when combined with REVEAL scores and CMR. Despite strong correlations with RHC, the AI-based model was limited by its inability to fully account for extreme pathologic abnormalities and trabeculation variability, highlighting the need for ongoing training and model refinement. Both studies emphasize the potential of AI-enhanced CMR in predicting PAH outcomes, though further prospective external validation and broader population testing are required.

### 3.6 Computed Tomography and AI Enhancement

Accurate phenotyping of PH subgroups remains clinically challenging due to significant phenotypic overlaps, particularly between Group 1 (PAH) and Group 3 (PH due to lung diseases, PH-LD) [54]. This underscores the critical role of quantitative lung parenchymal assessment in differential diagnosis. While conventional evaluation relied on semi-quantitative density scoring by clinicians—a method limited by interobserver variability—emerging AI platforms now leverage multi-omics approaches. Advanced texture feature extraction and supervised classification algorithms enable automated quantification of parenchymal remodeling patterns (e.g., fibrotic burden, microvascular distortion). These computational phenotyping tools not only refine diagnostic precision but also establish continuous severity indices for longitudinal monitoring, marking a paradigm shift from qualitative scoring to data-driven precision pulmonology.

While CMR offers detailed functional and tissue-level insights, CT provides complementary high-resolution visualization of pulmonary vasculature and lung parenchyma, enabling assessment of vascular remodeling, thromboembolic disease, and associated pulmonary pathology. CT plays a pivotal role in evaluating unexplained dyspnea or suspected/confirmed PH. Key CT imaging features indicative of PH include PA enlargement (diameter ≥30 mm), a PA-to-aorta ratio >0.9, and right ventricular/atrial dilatation [27]. A diagnostic triad combining PA diameter ≥30 mm, right ventricular outflow tract (RVOT) wall thickness ≥6 mm, and septal bowing ≥140° (or RV-to-LV ratio ≥1) demonstrates high predictive accuracy for PH [55]. Non-contrast chest CT helps identify PH etiology by detecting parenchymal abnormalities or features suggestive of pulmonary veno-occlusive disease/pulmonary capillary hemangiomatosis (PVOD/PCH), such as ground-glass opacities, interlobular septal thickening, and lymphadenopathy. In addition, computed tomography pulmonary angiography (CTPA) is used primarily to detect chronic thromboembolic pulmonary hypertension (CTEPH) [56], revealing features like vascular filling defects, thrombus attachment, and PA retraction or dilatation. CTPA can also identify other cardiovascular abnormalities, including intracardiac shunts and pulmonary arteriovenous malformations. While CTPA has a sensitivity of 76% and specificity of 96% for CTEPH diagnosis, its accuracy improves with high-quality multi-detector CT and expert interpretation [57,58,59].

However, one of the limitations of traditional CT assessment in PH is its semi-qualitative nature, relying heavily on visual interpretation [60]. Therefore, AI-assisted cardiac CT provides an efficient and accurate approach to obtaining critical metrics for diagnosing, phenotyping, and predicting outcomes in PH. AI tools are particularly effective in identifying and quantifying changes in lung parenchyma. While AI demonstrates high diagnostic accuracy in detecting acute pulmonary embolism, its performance is less reliable when it comes to chronic pulmonary embolism [5]. Quantitative computed tomography (QCT) has evolved into an AI-enabled framework that integrates automated lung segmentation, radiomic biomarker extraction, and predictive modeling. By combining high-dimensional imaging with clinical and hemodynamic data, QCT enables early detection, precision phenotyping, and dynamic risk assessment in PH [57].

This section will show AI improves the application of AI in PH from shallow to deep in three dimensions.

#### 3.6.1 Cardiac Segmentation

Semantic segmentation in medical imaging is the classification and delineation of pixels/voxels into regions representing anatomical structures. This identifies and quantifies morphological features [58,61]. Automated segmentation algorithms constitute the foundational technical layer in medical image analysis, executing pixel- and voxel-level classification to delineate cardiovascular anatomical structures (e.g. right ventricular myocardium, pulmonary arterial tree). This process generates morphometric biomarkers including biventricular mass-volume ratios, ejection fraction dynamics, and pulmonary arterial diameter gradients – parameters that form the cornerstone of critical clinical functions.

Segmentation tools have been developed to identify individual structures for specific diagnostic purposes. The dice similarity coefficient (DSC) is a widely used overlap metric, with scores ranging from 0 to 1 representing no or perfect overlap between the AI-generated segmentation and the ground truth, respectively. Yuan et al. [62] developed PA-Net, a 2D network specifically designed for pulmonary artery segmentation in CTPA images to aid in pulmonary embolism diagnosis. Their approach demonstrated superior accuracy compared to other advanced segmentation tools, achieving a Dice similarity coefficient (DSC) score of 0.938, outperforming manual segmentation.

The left atrial volume indexed to body surface area (LAVI) is a clinically significant structural parameter that serves as a validated surrogate marker for chronically elevated left ventricular diastolic pressure (LVDP), reflecting long-term hemodynamic remodeling in cardiovascular pathophysiology [63]. Aquino et al. [64] proposed a method for left atrium segmentation in multiphase cardiac CTs using a 3D image-to-image network combined with a conditional variational autoencoder (cVAE). Their approach was tested on 55 patients scheduled for ablation due to atrial fibrillation and undergoing CT coronary angiography. The AI-derived left atrial volume measurements closely matched manual ones, while completing the process in less than half the time.

UNet is a state-of-the-art CNN widely used for segmentation that excels in image segmentation by using an encoder – decoder architecture to efficiently capture context and spatial information. Recent advancements in multi-structure cardiac segmentation via nnU-Net architectures demonstrate bifurcating technical pathways. Sharkey et al. [65] pioneered a two-stage nnU-Net framework for CTPA imaging, specifically optimized for PH cohorts, achieving mean DSC >0.85 across four-chamber views, aorta, and pulmonary arteries. Notably, reduced accuracy in right ventricular hypertrophy segmentation (DSC 0.58) mirrored established radiologist variability, highlighting intrinsic anatomical complexity. Validated across 1333 patients with PH or pulmonary embolism, this remains the sole PH-tailored tool enabling phenotype-agnostic deployment. Contrastingly, Chen et al. [66] extended substructure granularity to 19 components in lung cancer cohorts through memory-optimized multimodel nnU-Net ensembles, attaining 94% clinical acceptability for radiotherapy planning despite differing disease contexts. These diverging approaches underscore nnU-Net’s adaptability: Sharkey’s workflow prioritizes PH-specific pathophysiological relevance, while Chen’s paradigm maximizes anatomical comprehensiveness for therapeutic applications.

AI-driven cardiac CT segmentation enables automated quantification of precise morphometric biomarkers (e.g., ventricular volumetry, pulmonary arterial metrics) critical for PH management. These DL frameworks, exemplified by nnU-Net-based implementations, demonstrate sub-5% inter-observer variability in clinical validation, outperforming manual assessments in both diagnostic accuracy (AUC >0.92) and workflow efficiency (processing time <90 seconds per scan). Integrated with radiomic feature analysis, they facilitate phenotype stratification, treatment response monitoring, and prognostic modeling – transforming CT imaging from qualitative evaluation to a predictive data source in PH care pathways.

#### 3.6.2 Lung Parenchymal Assessment

Accurate phenotyping of PH subgroups remains clinically challenging due to significant phenotypic overlaps, particularly between Group 1 (PAH) and Group 3 (PH-LD) [54]. This underscores the critical role of quantitative lung parenchymal assessment in differential diagnosis. While conventional evaluation relied on semi-quantitative density scoring by clinicians—a method limited by interobserver variability—emerging AI platforms now leverage multi-omics approaches. Advanced texture feature extraction and supervised classification algorithms enable automated quantification of parenchymal remodeling patterns (e.g., fibrotic burden, microvascular distortion). These computational phenotyping tools not only refine diagnostic precision but also establish continuous severity indices for longitudinal monitoring, marking a paradigm shift from qualitative scoring to data-driven precision pulmonology.

Touloumes et al. [67] introduced a CNN classification network for pulmonary fibrosis diagnosis. Trained on 3600 CT scans and fine-tuned in an external US cohort, the model had an AUC of 0.997 with high sensitivity and specificity (91.3 and 95.3%) in both low and high pulmonary fibrosis prevalence populations. However, this model does not directly quantify disease burden, a limitation of classification models.

Sharkey et al. [68] developed an integrative AI framework combining nnU-Net-based lung segmentation (DSC 0.99) and DenseNet-121 texture classification to assess pulmonary parenchymal remodeling in PH. Validated across idiopathic PAH and PH-LD cohorts, the system quantifies five distinct parenchymal patterns, achieving AUCs of 0.94–0.95 in correlating radiographic features with disease severity and DLCO metrics. Crucially, AI-derived fibrosis burden demonstrated prognostic value as an independent mortality predictor, advancing beyond conventional qualitative assessments to enable objective phenotyping in PH management. This suggests utility in disease identification, quantification and lung function assessment.

ML and DL algorithms demonstrate sub-millimeter precision in detecting fibrotic, emphysematous, and microvascular lung parenchymal remodeling patterns. DL architectures integrating three-dimensional CNN segmentation with radiomic feature extraction achieve high accuracy in distinguishing PH subtypes (PAH vs. PH-LD).

#### 3.6.3 Diagnosis, Classification and Prognostication

AI-enabled vascular analysis addresses fundamental challenges in PH diagnostics by overcoming human limitations in detecting early-stage vascular remodeling. Advanced segmentation algorithms provide precise arteriovenous mapping and quantitative vascular morphometry, enabling systematic identification of microvascular abnormalities and objective severity stratification. This computational approach transforms traditional subjective assessments into standardized biomarkers, proving particularly valuable for detecting subclinical vascular pathology and monitoring therapeutic responses across PH phenotypes.

Recent advancements in AI-driven vascular morphometry demonstrate transformative potential for PH subtyping, pathobiology characterization and diagnosis. Nardelli et al.’s [69] 3D CNN-graph cut architecture achieves cross-protocol artery-vein differentiation with 89–94% accuracy across non-contrast and CTEPH contrast-enhanced CT scans, overcoming traditional contrast dependency limitations. Subsequent applications reveal distinct remodeling signatures: PAH cohorts exhibit small vessel pruning (42% reduction vs. controls) with paradoxically increased large vessel volumes, while exercise PH shows analogous vascular attrition patterns suggesting incipient remodeling [70]. These automated quantifications of tortuosity indices and bifurcation geometry establish quantitative imaging biomarkers for detecting (1) early microvascular loss preceding hemodynamic thresholds and (2) phenotype-specific remodeling trajectories, effectively bridging radiographic features with vascular pathomechanisms in PH research. Melzig et al. [71] investigated the use of automated 3D volumetry of central pulmonary arteries from CTPA images, combined with echocardiographic data, to predict mPAP and diagnose PH. What's more, Zhang et al. [72] developed a fully automated framework for diagnosing PH using CTPA images.

Xi et al. [73] developed PerAIDE, an AI-driven model for quantifying pulmonary perfusion using dual-energy computed tomography pulmonary angiography (DE-CTPA), specifically for patients with chronic pulmonary thromboembolism (CPE). The goal was to offer a noninvasive, automated tool for assessing pulmonary perfusion blood volume (PBV) and to distinguish between chronic thromboembolic pulmonary disease (CTEPD) and CTEPH. The system used a U-Net architecture for lung segmentation and adaptive thresholding to classify regions of normal, reduced, and defective perfusion. Data from 183 patients (32 with CTEPD, 151 with CTEPH) demonstrated that PerAIDE showed high correlation with manual radiologist scoring (ICC = 0.778), reduced analysis time significantly (31 s vs. 15 min), and AUC of 0.809 in differentiating CTEPH from CTEPD. Key findings included a higher proportion of perfusion defects in CTEPH (0.35 vs. 0.29) and a correlation between perfusion defects and hemodynamic parameters, including mPAP and PVR. The limitations of the study include its single-center design, limited external validation, and the need for incorporating lobar and segmental quantification for better therapeutic guidance. These findings highlight PerAIDE’s potential in clinical risk stratification and treatment monitoring post-intervention.

Approximately 2–3% of acute pulmonary embolisms develop into CTEPH and with interventions such as PEA available for those with chronic thromboembolic disease (CTED). Therefore, identification of both acute and chronic pulmonary embolism is a clinically relevant endeavour. CTEPH can be misdiagnosed due to its nonspecific symptoms and reliance on specialized imaging modalities like ventilation/perfusion (V/Q) scans and CTA. However, even AI tools do not have high diagnostic accuracy in detecting in chronic pulmonary embolism. That is what the future development of AI-enabled the tools of CTEPH diagnosis should be focused [74].

Dwivedi et al. [75] developed a CT-based AI model for quantifying lung fibrosis and predicting survival in PH patients, aiming to improve prognostication beyond traditional visual scoring methods. The study involved 521 PH patients (median age 67, 52% women) who underwent CT pulmonary angiography (CTPA). An AI model was applied to quantify lung fibrosis and assess its association with patient mortality. The study found that AI-quantified fibrosis was an independent prognostic marker for mortality, with a hazard ratio of 1.03 (*p* = 0.004). Combining AI-quantified fibrosis with radiologic scoring improved survival predictions, yielding a C-index of 0.67, compared to 0.61 for radiologic scoring alone. Key findings suggest that AI can detect minor fibrosis in patients scored as having no fibrosis, providing more accurate survival risk assessment. However, the study’s limitations include its retrospective design, potential inter-observer variability in radiologic scoring, and the multicenter study, which may limit the generalizability of the findings. These results highlight the potential of AI-driven fibrosis quantification in improving clinical outcomes and treatment strategies for PH patients. Table 5 (Ref. [65,68,69,70,71,72,73,75]) summarizes the key findings related to the application of AI in CT as discussed in this section.

**Table 5.** **Summary of AI in CT applications in PH**.

Author (Year)	Population & PH-group	Threshold & Reference standard	Study design & Validation	Model	Key findings & Performance	Limitations
Sharkey et al. (2022) [65]	**Pop:** Train/Val/Test: 200 internal patients; Ext Test: 20 patients. Failure analysis in 1333 mixed disease patients. **Group:** Cardiac & Vessel Segmentation on CTPA	**Thres:** N/A (Segmentation). **Ref: **Manual Segmentation by Radiologists	**Design: **Retrospective Model Development. **Val: **Internal (Hold-out) & External Testing	3D U-Net (Two-stage Cascade)	The model achieved high Dice scores (>0.90 for most structures) in both internal and external cohorts, with RV myocardial volume showing strong correlation with mPAP (**γ **= 0.70), comparable to or better than manual metrics	1. Relatively small external test set (n = 20), limiting assessment of scanner variability 2. Lower segmentation performance for the right ventricular myocardium 3. Reliance on expert manual segmentation as ground truth
Sharkey et al. (2023) [68]	**Pop: **122 Precapillary PH patients (83 Train, 17 Val, 10 Internal Test, 12 External Test). **Group: **Lung Texture Classification (Normal, Ground Glass, Honeycombing, Emphysema)	**Thres: **5 Texture Classes (Fleischner Society Terms). **Ref: **Expert Radiologist Segmentation & DLCO	**Design:** Retrospective Development & Validation. **Val:** Internal (Hold-out) & External (Independent Center)	DenseNet-121 (Patch-based 2.5D CNN).	The model achieved excellent classification performance with micro-average AUCs of 0.95 (Internal) and 0.94 (External). AI-quantified lung disease burden showed strong correlation with functional metrics like DLCO (*r* = 0.79) and matched radiologist severity scoring	1. Small sample size, particularly the external test set (n = 12), limiting robust assessment of generalizability 2. Potential label bias arising from reliance on annotations 3. Use of high-quality thin-slice scans, which may limit performance generalizability
Nardelli et al. (2018) [69]	**Pop: **Train: 25 non-contrast CTs (COPDGene); Val/Test: 18 subjects (COPDGene) + 33 contrast CTs (PH/CTEPH cohort). **Group:** Pulmonary Artery-Vein Classification	**Thres: **Artery vs. Vein Classification. **Ref:** Manual Labeling by Experts	**Design:** Algorithm Development & Cross-modality Validation. **Val: **Internal (COPDGene) & External (PH/CTEPH Cohort)	3D CNN + Graph-Cuts Optimization	The method achieved 94% accuracy on non-contrast CTs and generalized well to contrast-enhanced CTs (accuracy 89%, sensitivity 93%) without retraining. It significantly outperformed Random Forests and standard vessel enhancement filters in differentiating arteries from veins	1. Dependence on pre-segmentation of vessel particles, with errors propagating to downstream classification 2. Degraded performance in distal vessels (<2 mm) due to partial volume effects 3. Increased computational cost from graph-cut optimization compared with CNN inference 4. Restriction to specific scanner types, limiting generalizability
Rahaghi et al. (2021) [70]	**Pop: **Retrospective: 91 subjects (42 PAH, 12 Exercise-PH, 37 Controls) with paired CT and RHC. **Group: **Arterial/Venous Morphometry (Pruning & Tortuosity)	**Thres: **mPAP >20 mmHg (PAH) / Exercise mPAP Rise (ePH). **Ref: **RHC	**Design: **Retrospective Cohort Analysis. **Val: **Clinical Correlation (Hemodynamics)	Automated Artery-Vein Separation (CNN + Scale-space Particles)	PAH patients showed reduced arterial and venous small vessel volume (*p *< 0.0001) and increased arterial tortuosity. Interestingly, ePH patients exhibited venous pruning similar to PAH but lacked significant arterial pruning, suggesting venous remodeling may precede arterial changes in early disease	1. Control group composed of symptomatic patients with dyspnea rather than healthy volunteers 2. Long time interval between CT and RHC 3. Potential confounding effects of vasodilator therapy in PAH patients on morphologic measurements
Melzig et al. (2019) [71]	**Pop: **70 patients with suspected PH (mean age 66.7) undergoing CTPA, Echo, and RHC. **Group: **PH Detection (MPA Volumetry)	**Thres:** Predicted mPAP >25.8 mmHg. **Ref: **RHC	**Design:** Retrospective Diagnostic Accuracy Study. **Val:** Internal Comparison with Invasive Hemodynamics	Automated 3D Volumetry (Model-based Segmentation).	Automated 3D MPA volume correlated strongly with mPAP (***γ ***= 0.76), outperforming 2D diameters. Combining 3D volumetry with echocardiographic PASP yielded the highest accuracy (AUC 0.94, ***γ ***= 0.89), with 86% sensitivity and 93% specificity	1. Retrospective study design with a small, single-center cohort of patients already suspected of PH, 2. Model-based segmentation requiring manual initialization in some cases, lacking a fully end-to-end DL workflow 3. Dependence on simultaneous availability of high-quality CTPA and echocardiography data
Zhang et al. (2023) [72]	**Pop:** 55 PH patients (44 Training, 11 Testing) undergoing CTPA and RHC. **Group:** Pulmonary Artery Pressure Estimation	**Thres:** mPAP >40 mmHg, sPAP >55 mmHg (Classification). **Ref:** RHC	**Design:** Retrospective Single-center Study. **Val:** Internal (Hold-out Test Set)	nnU-Net (Segmentation) + ML Regression/Classification.	The automated framework achieved high agreement with RHC, with an ICC of 0.934 for mPAP prediction. For classifying severe PH (mPAP >40 mmHg), the model achieved an AUC of 0.911. Automated segmentation improved Dice scores to 88.2% compared to baseline	1. Very small sample size (n = 55) and single-center study design 2. Reliance on 1D/2D morphological features (e.g., diameters) rather than volumetric or textural information 3. Retrospective study design with potential selection bias
Xi et al. (2025) [73]	Pop: 183 patients (151 CTEPH, 32 CTEPD) from a single center. **Group: **CTEPH (Perfusion Quantification)	**Thres:** Perfusion Patterns (Normal/Reduced/Defective). **Ref:** Radiologist Assessment/RHC	**Design:** Prospective Observational Study. **Val:** Internal (ICC with Radiologists)	PerAIDE (U-Net based AI System)	PerAIDE significantly reduced analysis time (31 s vs. 15 min) and showed high agreement with radiologists (ICC = 0.78). Perfusion defects were greater in CTEPH than CTEPD (0.35 vs. 0.29) and correlated with PVR (***γ ***= 0.43)	1. Single-center study design, requiring multicenter validation 2. AI segmentation limited to whole-lung analysis, lacking lobar or segmental granularity 3. Absence of integration of morphological imaging data and long-term clinical outcomes
Dwivedi et al. (2024) [75]	**Pop: **521 patients (275 Training, 246 External Test) with IPAH or PH-LD. **Group:** Lung Fibrosis Quantification & Prognostication	**Thres:** Fibrosis Severity (Mild/Mod/Severe). **Ref:** Radiologic Scoring/Survival (Mortality)	**Design:** Retrospective Multicenter Study. **Val:** External Testing (37 Hospitals)	DenseNet121 (Patch-based DL)	AI-quantified fibrosis (combined with radiologic scoring) significantly improved survival prediction compared to radiologic scoring alone (C-index 0.67 vs. 0.61, ***p ***< 0.001). It provided reproducible quantification of ground glass and reticulation	1. Retrospective study design with potential selection bias due to inclusion of only patients with thin-section CTPA 2. Demographic and image quality differences between training and external cohorts 3. Variability in breath-holding and contrast timing

CT, computed tomography; CTPA, computed tomography pulmonary angiography; DLCO, diffusing capacity of the lung for carbon monoxide; COPDGene, Chronic Obstructive Pulmonary Disease Genetic Epidemiology study; CTEPH, chronic thromboembolic pulmonary hypertension; sPAP, systolic pulmonary artery pressure; CTEPD, chronic thromboembolic pulmonary disease.

## 4. Discussion

### 4.1 Diagnostic Accuracy Across Modalities

The diagnostic landscape of AI in PH is characterized by a distinct hierarchy of performance, evolving from single-modality screening to holistic multimodal integration. Individual modalities have achieved impressive milestones, such as DL-enhanced ECG detecting subclinical electrical remodeling with high sensitivity and CXR models utilizing texture analysis to identify microvascular pruning. However, they remain constrained by inherent limitations, including specificity “traps” in low-prevalence populations and ground-truth variability in echocardiography. Advanced imaging modalities like CT and CMR offer the highest single-modality precision through “virtual hemodynamics”, yet their accessibility is limited. Consequently, the most significant recent breakthrough lies in Multimodal Fusion (MMF) architectures. Evidence from 2024–2025 demonstrates that fusing electrical (ECG), anatomical (CXR/CT), and functional (Echo) data enables AI to capture the complex, multi-systemic nature of PH. This integration elevates diagnostic accuracy to near-perfect levels and significantly outperforms any single modality in phenotype differentiation.

### 4.2 Limitations and Challenges

Despite the transformative potential of AI in PH, significant technical and methodological barriers impede its translation from “in silico” validation to widespread clinical adoption. These challenges are not merely logistical but stem from fundamental conflicts between data science methodologies and clinical realities.

#### 4.2.1 The “Ground Truth” Dilemma and Label Imperfection—the Trade-Off Between Label Accuracy and Dataset Size

Many large-scale studies train AI models using echocardiographic parameters (e.g., TR velocity > 2.8 m/s) as the ground truth. Since echocardiography itself has significant inter-observer variability and correlates imperfectly with invasive hemodynamics, AI models trained on this data inherently learn to reproduce human estimation errors (“learning the noise”) rather than the true physiological state. Conversely, models trained on the gold standard—RHC—suffer from severe selection bias. RHC datasets typically represent a “sick” population with high pre-test probability. However, they often lack the healthy controls or mild disease phenotypes encountered in primary care screening. This discrepancy limits the utility of RHC-trained models when deployed in general populations.

#### 4.2.2 Generalizability and the “Prevalence Trap”—Most Validated Algorithms Originate From Single-Center Retrospective Cohorts With Artificially Balanced Datasets

When these high-performing models (AUC >0.90) are deployed in real-world screening settings where PH prevalence is low (< 1%), the positive predictive value drops precipitously. An algorithm with 90% specificity will generate 10 false positives for every true positive in a low-prevalence population. Such results could potentially overwhelm specialized centers with unnecessary referrals and expose patients to risks from invasive confirmatory tests. Current training datasets often lack diversity in race, age, and BMI. AI models have been shown to use demographic shortcuts (e.g., recognizing age or sex markers in CXR) rather than pathological features to make predictions, leading to algorithmic bias and reduced performance in underrepresented groups (e.g., pediatric PH or specific ethnic minorities).

#### 4.2.3 Data Harmonization and Domain Shift—the Implementation of AI Faces Critical Limitations in Interoperability

Medical imaging data are highly sensitive to acquisition protocols. A model trained on GE MRI scanners may fail when applied to Siemens or Philips data due to subtle differences in signal processing and pixel distributions—a phenomenon known as “domain shift.” The proliferation of proprietary data formats from wearable technologies further exacerbates these inconsistencies. PH data remains siloed in center-specific warehouses due to privacy regulations (GDPR/HIPAA). While federated learning offers a theoretical solution by training models across decentralized data without sharing patient records, the infrastructure for such collaboration is currently immature in most hospital systems.

#### 4.2.4 The “Black Box” Problem and Shortcut Learning—the Opacity of DL Models Remains a Critical Barrier to Clinical Trust

Unlike traditional regression models, DL models do not provide a clear rationale for their decisions. There is a risk of “shortcut learning”, where the AI focuses on confounding features (e.g., a pacemaker lead or a specific text marker on CXR) rather than disease pathology. Although techniques such as Grad-CAM attempt to visualize AI attention, they often provide only coarse localization. In high-stakes decisions, like listing a patient for transplant, clinicians require more than a heat map; they need interpretable biomarkers that align with physiological principles.

## 5. Future Directions

To address current limitations, future research directions should focus on advancing methodological transparency, data governance, and clinical integration.

Advancing Explainable AI (XAI) is essential to bridge the gap between “black-box” algorithms and clinical trust. Integrating interpretability techniques, such as SHAP and Grad-CAM, allows clinicians to visualize the physiological features driving AI decisions. Furthermore, adopting attention-based architectures such as Vision Transformers can align AI focus with pathophysiological mechanisms, transforming models into transparent, verifiable tools.

Beyond model interpretability, addressing data privacy and generalizability remains a central challenge for large-scale clinical deployment. Federated learning [76], which enables decentralized model training across multiple institutions without direct data sharing, holds substantial promise for addressing data privacy concerns while supporting the development of generalizable AI models across diverse patient populations and institutional governance frameworks. In parallel, transfer learning strategies may further enhance model robustness by allowing pre-trained algorithms to be adapted to new and heterogeneous datasets, improving performance across varied clinical settings. The development of standardized, integrated diagnostic platforms that combine clinical parameters, laboratory biomarkers, multimodal imaging data, and molecular or genomic profiles will be essential for detecting subclinical disease manifestations, refining PH phenotyping, and supporting precision therapeutic decision-making. To ensure these advances translate into reliable clinical tools, multicenter prospective trials remain critical for validating AI-driven diagnostic tools across diverse populations and care environments, ensuring reproducibility and clinical reliability.

Looking further ahead, the next phase of innovation is likely to be driven by synergistic, human-centered AI ecosystems. Multimodal Fusion [77], Generative AI, and Collaborative Intelligence. Specifically, the development of intermediate fusion architectures that synthesize discordant signals from ECG, imaging, and clinical data is paramount for achieving expert-level phenotypic precision. Zhao et al. [78] developed a novel framework (MMF-PH) based on CXR–centered multimodal data fusion, incorporating electrocardiographic and echocardiographic data (textual and tabular) to enhance non-invasive PH screening. Validated against the gold standard RHC in a large cohort of nearly 3000 patients, the model demonstrated significantly superior specificity and robustness compared to unimodal TTE. These findings provide compelling evidence that heterogeneous data fusion effectively overcomes the diagnostic limitations of single-modality approaches by capturing complementary hemodynamic and electrophysiological pathological features. In parallel, integrating Large Language Model (LLM) agents [79] to autonomously mine unstructured electronic health records (EHRs) offers a scalable solution for identifying undiagnosed patients (“computational phenotyping”) and recovering missing hemodynamic data. Finally, effective translation into real-world practice will require seamless integration of computational tools into existing clinical workflows through decision-support interfaces that provide evidence-based recommendations while preserving physician autonomy, diagnostic transparency, and individualized patient management.

## 6. Conclusion

This review demonstrates the current state and clinical potential of AI applications in PH imaging diagnostics across multiple modalities. Computer-assisted cardiovascular imaging analysis has achieved diagnostic performance metrics across ECG, CXR, echocardiography, CMR, and CT platforms, with demonstrated utility in automated image interpretation, risk stratification, and subclinical disease detection. Despite these advances, significant implementation barriers persist, including limited external validation across diverse patient populations, insufficient algorithm interpretability for clinical decision-making, and inadequate integration frameworks for existing diagnostic workflows. Translation into routine clinical practice requires multicenter prospective validation studies, standardized regulatory pathways for algorithm approval, and evidence-based integration protocols that preserve physician oversight while enhancing diagnostic precision. Standardized multimodal frameworks incorporating imaging biomarkers with hemodynamic and laboratory parameters must be established to ensure equitable healthcare access across diverse clinical settings. The clinical impact of these technologies will ultimately depend on sustained interdisciplinary collaboration between cardiovascular imaging specialists, clinicians, and biomedical engineers to develop validated diagnostic tools that demonstrate measurable improvements in patient outcomes and healthcare delivery efficiency in PH management.

## Appendix

See Table 6.

**Table 6.** **Risk of bias assessment of included studies using PROBAST**.

Study (Modality)	Participants	Predictors	Outcome	Analysis	Overall
**ECG Studies**					
Sawada et al., 2019 [16]	Unclear	Low	Low	High	High
DuBrock et al., 2024 [19]	Low	Low	Low	Low	Low
Kwon et al., 2020 [20]	Low	Low	High	Unclear	High
Nemati et al., 2024 [23]	High	Low	High	High	High
**CXR Studies**					
Liu et al., 2025 [22]	Low	Low	High	Low	High
Ueda et al., 2023 [26]	Low	Low	High	Low	High
Kusunose et al., 2020 [30]	Low	Low	Low	High	High
Kusunose et al., 2022 [31]	Low	Low	High	Low	High
Zou et al., 2020 [32]	High	Low	High	Low	High
Imai et al., 2024 [33]	High	Low	High	High	High
White et al., 2025 [34]	Unclear	Low	High	High	High
Kıvrak et al., 2023 [35]	Low	Low	High	Low	High
**Echo Studies**					
Zhang et al., 2018 [40]	Unclear	Low	High	High	High
Anand et al., 2024 [41]	Low	Low	Low	High	High
Sun et al., 2024 [42]	Low	Low	High	Low	High
Hirata et al., 2024 [43]	Low	Low	Low	High	High
Fortmeier et al., 2022 [44]	Low	Low	Low	Low	Low
**CMR Studies**					
Dawes et al., 2017 [47]	Low	Low	Low	High	High
Attard et al., 2019 [48]	Low	Low	Low	Unclear	Unclear
Duan et al., 2019 [49]	Low	Low	Low	Unclear	Unclear
Swift et al., 2021 [50]	Low	Low	Low	High	High
Wang et al., 2024 [51]	Low	Low	Low	Low	Low
Zeng et al., 2025 [52]	High	Low	Low	High	High
Alabed et al., 2022 (DH) [53]	Low	Low	Low	High	High
Alabed et al., 2022 (Rad) [53]	Low	Low	Low	Low	Low
**CT Studies**					
Sharkey et al., 2022 [65]	Low	Low	Low	High	High
Sharkey et al., 2023 [68]	Low	Low	High	High	High
Nardelli et al., 2018 [69]	High	Low	High	High	High
Rahaghi et al., 2021 [70]	Low	Low	Low	High	High
Melzig et al., 2019 [71]	Low	Low	Low	High	High
Zhang et al., 2023 [72]	Unclear	Low	Low	High	High
Xi et al., 2025 [73]	Low	Low	Low	High	High
Dwivedi et al., 2024 [75]	Low	Low	Low	Low	Low

Note: ROB, Risk of Bias.
